# The experience and impact of stigma in Saudi people with a mood disorder

**DOI:** 10.1186/s12991-018-0221-3

**Published:** 2018-11-27

**Authors:** Deemah AlAteeq, Abdullah AlDaoud, Ahmad AlHadi, Hanoof AlKhalaf, Roumen Milev

**Affiliations:** 1 Department of Psychiatry, College of Medicine, King Saud University, P.O. box 92949, Riyadh, 11683 Saudi Arabia; 2 SABIC Psychological Health Research & Applications Chair, College of Medicine, King Saud University, Riyadh, Saudi Arabia; 3 Department of Psychiatry, Queen’s University, Kingston, ON Canada

**Keywords:** Stigma, Bipolar disorder, Depression, Cross-cultural comparison, Saudi Arabia, Inventory of Stigmatizing Experiences

## Abstract

**Background:**

Stigma plays a powerful role in an individual’s attitude towards mental illness and in their seeking psychiatric and psychological services. Assessing stigma from the perspective of people with mood disorders is important as these disorders have been ranked as major causes of disability.

**Objectives:**

To determine the extent and impact of stigma experiences in Saudi patients with depression and bipolar disorder, and to examine stigma experiences across cultures.

**Method:**

Ninety-three individuals with a mood disorder were interviewed at King Saud University Medical City using the Inventory of Stigmatizing Experiences (ISE).

**Results:**

We detected no significant differences in experiences of stigma or stigma impact in patients with bipolar vs. depressive disorder. However, over 50% of respondents reported trying to hide their mental illness from others to avoiding situations that might cause them to feel stigmatized. In comparison with a Canadian population, the Saudi participants in this study scored significantly lower on the ISE, which might be due to cultural differences.

**Conclusion:**

More than half of the Saudi participants with a mood disorder reported avoiding situations that might be potentially stigmatizing. There are higher levels of stigma in Canada and Korea than in Saudi Arabia. Our results suggest that cultural differences and family involvement in patient care can significantly impact self-stigmatization. The ISE is a highly reliable instrument across cultures.

## Introduction

The World Health Organization ranked major depression as the fourth most common cause of disability-adjusted life years. Additionally, bipolar disorder ranked as the sixth most common cause of disability [1]. However, stigmatization of individuals with mental illness is widespread and poses a major barrier to treatment [2, 3]. It also leads to a decrease in compliance with therapeutic interventions, early termination of treatment [4], and added difficulties in the patient’s daily life [5].

Many aspects of the stigmatization of mental illness are culture-specific. Relative to Western countries, in developing countries in Asia and in the Muslim community, there is a widespread tendency to stigmatize and discriminate against people with mental illness. Efforts to maintain social distance from people with mental illness sometimes result from considering them to be dangerous and aggressive. Moreover, supernatural, religious, and magical approaches to mental illness are predominant [6, 7]. The core belief regarding health and mental health in the Muslim community is centered around destiny, in which the predominant attitude is positive acceptance of God’s will and high levels of optimism towards healing [8–13].

In particular, in Saudi Arabia, qualitative research conducted in half of the Primary Health Care centers in Al-Khobar in 2012 suggested several reasons for stigma towards psychiatric disorders within the Saudi community. The most frequently mentioned reasons were traditions, cultural norms, the way people were raised, and a lack of community awareness. Other reasons included a fear of aggression and violence from psychiatric patients and a fear of the side effects of psychiatric medications. Psychiatric diseases were also widely reported to be either hereditary or incurable [14].

The majority of studies have assessed the knowledge and attitudes of the general public, but the perspectives of people with a mental illness have not yet been assessed in Arab communities. To our knowledge, no studies have addressed the social stigma that patients experience in Saudi Arabia and quantitatively compared it to the experiences of patients in other cultures. Most of the literature regarding stigma towards people with mental illness relates to people with more severe symptoms, such as those associated with schizophrenia and other psychotic disorders [14–16].

This study aimed to determine the impact and the extent of social stigma on patients in Saudi Arabia with mood disorders and to compare the results with the findings of the Canadian and Korean studies as a part of a multicenter international research project [17, 18]. We also investigated the effect of demographic characteristics on the experience of stigma among those with bipolar disorder or depression in Saudi Arabia. This could improve our knowledge about the impact of Saudi culture on patients and the extent to which patients have experienced stigma, including in relation to other countries, and could thereby highlight the importance of client-centered anti-stigma programs for patients with a mood disorder.

## Methods

### Participants

This was a cross-sectional study. Patients with a mood disorder were interviewed using a valid and reliable questionnaire. The study sample was recruited using convenience sampling of patient records of the psychiatric outpatient clinic and from the psychiatric inpatient ward at King Saud University Medical City, Riyadh, between August 2013 and September 2015. As there have been no epidemiological studies investigating people with a mood disorder who reported their experiences of stigma, there were no prevalence data on which to base sample size estimates. Although refusals to participate were not formally tracked, almost everyone who was asked to be interviewed agreed to participate in the study. Thus, we estimated that the response rate was approximately 85%. There were no restrictions regarding sex, or ethnicity. The inclusion criteria were as follows: participants over 18 years old, with a current diagnosis of depression or bipolar disorder (either type I or II), and who met diagnostic criteria according to DSM-IV-TR. The exclusion criteria were patients with severe depression and psychosis as well as strong suicidal inclinations or tendencies.

### Measures

To assess their experiences of stigma, patients were asked to answer all the items of the Inventory of Stigmatizing Experiences [19]. In the ISE, stigma is defined as “negative feelings people have towards people with a mental illness”. The questionnaire consists of two subscales, as follows: the Stigma Experiences Scale, which includes ten items that measure both the frequency and prevalence of negative experiences of stigma, and the Stigma Impact Scale, which includes seven items that measure the intensity of the psychosocial impact.

Regarding scoring of the Stigma Experiences Scale, 13 of the 15 items may be answered “yes”, “unsure”, or “no”, while the other 2 items may be answered “never”, “rarely”, “sometimes”, “often”, or “always”. All items were recorded to reflect the presence or absence of stigma, and reverse scoring was used for some items. Meanwhile, the Stigma Impact Scale consists of 7 questions that are rated on a scale from 0 (lowest possible score) to 10 (highest possible score). Four items are used to rate the degree to which stigma negatively impacts the respondent’s quality of life, social contacts, family relations, and self-esteem. The remaining 3 items on the Stigma Impact Scale are used to rate the degree to which stigma negatively impacts the respondent’s family’s quality of life, social contacts, and family relations. The extent of stigmatizing experiences was determined by calculating the mean of the total score on the Stigma Experiences Scale. The psychosocial impact of stigma on each group was determined by calculating the mean of the total score on the Stigma Impact Scale.

The ISE has previously been tested by Stuart et al. for reliability purposes in a heterogeneous sample of psychiatric outpatients. The reliability coefficients were high for both scales: 0.83 for the Stigma Experiences Scale and 0.91 for the Stigma Impact Scale [19]. To create an Arabic version of the inventory, the original questionnaire was first translated into Arabic by a member of the research team. To ensure that the translation was accurate, an individual who was not a member of the research team and who did not have access to the English version of the questionnaire back-translated the Arabic version into English. The back-translated version was then compared with the original English inventory, and necessary revisions were made.

### Procedure

The questionnaire was administered as an interview with a trained member of the research team. The interviews took place either at the clinic or on the psychiatric ward. To guarantee the accuracy of the patient’s self-reported psychiatric history and diagnosis status, their medical files were collected with his or her consent, and any relevant information was retrieved.

### Statistics

The socio-demographic and clinical characteristics of the groups are described using one- and two-way frequency distributions with proportions. The intergroup difference in the extent of stigmatizing experience was tested using an independent samples *t*-test (with stigma experience as the dependent variable and diagnostic group or nationality as the independent variable). The intergroup difference in psychosocial impact of stigma was determined using an independent samples *t*-test.

To determine the reliability of the ISE, reliability coefficients were calculated by measuring the percentage of endorsed correlation and item-rest correlations. The internal consistencies of the scales were assessed using the Kuder–Richardson Formula 20 (KR-20) reliability coefficient for the experiences scale, which was composed of binary items, and Cronbach’s alpha for the impact scale, which was composed of interval data. All analyses were conducted using the Statistical Package for Social Sciences, version 20.0 (SPSS 20; IBM Corp., Armonk, NY, USA) for Windows^®^.

## Results

### Socio-demographic and clinical characteristics

A total of 93 individuals completed the interview for the study. They were diagnosed with either bipolar disorder (50 individuals) or depression (43 individuals). Table 1 summarizes the socio-demographic and clinical characteristics of the patients by diagnostic group. The majority of our sample was female (67.7%), which is similar to Canada (59.3%) and Korea (70.6%). This can be explained by the higher lifetime prevalence of mood disorders in females compared to males, as reported in the National Comorbidity Survey Replication and Adolescent Supplement [20]. The majority of participants were unemployed (75.3%). Again, this is similar to the results for Canada (75.9%) and Korea (70.6%). Approximately half of the unemployed participants in our sample were unable to work due to psychiatric problems or other medical problems. Unemployment may lead to a new social identity that is stigmatized and associated with impaired well-being [21].

**Table 1:** Social characteristics of the participants (*N *=93)

Characteristic	Bipolar disorder (*n* = 50)	Depression (*n* = 43)	Total (*n* = 93)
Sex			
Male	19 (38.8%)	11 (25.6%)	30 (32.3%)
Female	31 (62.0%)	32 (74.4%)	63 (67.7%)
Age group			
29 years and younger	11 (22.0%)	4 (9.3%)	15 (16.1%)
30–39 years	18 (36.0%)	9 (20.9%)	27 (29%)
40–49 years	10 (20.0%)	12 (27.9%)	22 (23.7%)
50–59 years	7 (14.0%)	13 (30.2%)	20 (21.5%)
60–72 years	4 (8.0%)	5 (11.6%)	9 (9.7%)
Highest level of education			
Illiterate	0 (0%)	4 (9.3%)	4 (4.3%)
Elementary or intermediate school	7 (14.0%)	15 (34.9%)	22 (23.7%)
High school	12 (24.0%)	10 (23.3%)	22 (23.7%)
College or technical training	5 (10.0%)	4 (9.3%)	9 (9.7%)
University	21 (42.0%)	10 (23.3%)	31 (33.3%)
Postgraduate study	5 (10.0%)	0 (0%)	5 (5.4%)
Marital status			
Single	20 (40.0%)	6 (14.0%)	26 (28%)
Separated	1 (2.0%)	5 (11.6%)	6 (6.5%)
Widowed	3 (6.0%)	2 (4.7%)	5 (5.4%)
Divorced	3 (6.0%)	3 (7.0%)	6 (6.5%)
Married	23 (46.0%)	27 (62.8%)	50 (53.8%)
Living situation			
Alone	0 (0%)	1 (2.3%)	1 (1.1%)
With spouse	18 (36.0%)	27 (62.8%)	45 (48.4%)
With parents	24 (48.0%)	10 (23.3%)	34 (36.6%)
With another close relative	6 (12.0%)	3 (7.0%)	9 (9.7%)
Other	2 (4.0%)	2 (4.7%)	4 (4.3%)
Employment status			
Employed	17 (34.0%)	6 (14.0%)	23 (24.7%)
Not employed	33 (66.0%)	37 (86.0%)	70 (75.3%)
Nature of employment			
Unable to work due to psychiatric problems	10 (20.0%)	10 (23.3%)	20 (21.5%)
Unable to work due to medical problems	11 (22.0%)	15 (34.9%)	26 (28%)
Homemaker	3 (6.0%)	5 (11.6%)	8 (8.6%)
Retired	8 (16.0%)	6 (14.0%)	14 (15.1%)
Volunteer worker	1 (2.0%)	1 (2.3%)	2 (2.2%)
Full-time worker	17 (34.0%)	6 (14.0%)	23 (24.7%)
Mental health now compared to a year ago			
Better	38 (76.0%)	25 (58.1%)	63 (67.7%)
About the same	10 (20.0%)	12 (27.9%)	22 (23.7%)
Worse	2 (4.0%)	6 (14.0%)	8 (8.6%)
Age at symptom onset			
9–19 years	19 (38.0%)	16 (38.1%)	35 (38.0%)
20–29 years	21 (42.0%)	8 (19.0%)	29 (31.5%)
30–39 years	4 (8.0%)	12 (28.6%)	16 (17.4%)
40+ years	6 (12.0%)	6 (14.3%)	12 (13.0%)
Age at first treatment			
10–19 years	16 (32.0%)	8 (18.6%)	24 (25.8%)
20–29 years	23 (46.0%)	9 (20.9%)	32 (34.4%)
30–39 years	5 (10.0%)	12 (27.9%)	17 (18.3%)
40+ years	6 (12.0%)	14 (32.6%)	20 (21.5%)
Number of years between symptom onset and first treatment			
Under 1 year	30 (60.0%)	18 (42.9%)	48 (52.2%)
1–2 years	10 (20.0%)	5 (11.9%)	15 (16.3%)
3–5 years	8 (16.0%)	7 (16.7%)	15 (15.3%)
6–10 years	1 (2.0%)	5 (11.9%)	6 (6.5%)
10+ years	1 (2.0%)	7 (16.7%)	8 (8.7%)
Have come to accept their diagnosis			
No	5 (10.0%)	5 (11.6%)	10 (10.8%)
Yes	45 (90.0%)	38 (88.4%)	83 (89.2%)
Years between treatment initiation and the acceptance of their diagnosis			
Not accepted	5 (10%)	5 (11.6%)	10 (10.8%)
< 1 year	34 (68.0%)	31 (72.1%)	65 (69.9%)
1–5 years	8 (16 .0%)	4 (9.3%)	12 (12.9%)
6–10 years	1 (2.0%)	1 (2.3%)	2 (2.2%)
11–15 years	1 (2.0%)	2 (4.7%)	3 (3.2%)
21–25 years	1 (2.0%)	0 (0%)	1 (1.1%)
Ever hospitalized for a mental illness or suicide attempt			
Yes	36 (72.0%)	11 (25.6%)	47 (50.5%)
No	14 (28.0%)	32 (74.4%)	46 (49.5%)
Hospital use			
Ever hospitalized in a provincial psychiatric institution	1 (3.2%)	3 (37.5%)	4 (10.3%)
Ever hospitalized in a general hospital psychiatric unit	30 (96.8%)	5 (62.5%)	35 (89.7%)
Services used in the last year			
Voluntarily hospitalized in the last year	10 (27.0%)	2 (18.2%)	12 (25.0%)
Involuntarily hospitalized in the last year	27 (73.0%)	9 (81.8%)	36 (75.0%)
Use of outpatient community mental health programs in the last year			
Yes	29 (58.0%)	29 (67.4%)	58 (62.4%)
No	21 (42.0%)	14 (32.6%)	35 (37.6%)
Frequency of outpatient treatment (*N *=58)			
Weekly	2 (6.9%)	3 (10.3%)	5 (8.6%)
2–3 times per month	0 (0%)	1 (3.4%)	1 (1.7%)
Monthly	5 (17.2%)	0 (0%)	5 (8.6%)
Every 2–3 months	2 (6.9%)	5 (17.2%)	7 (12.1%)
1–2 times per year	20 (69.0%)	20 (69.0%)	40 (69.0%)

### Experience of stigma in Saudi Arabia

Table 2 summarizes the percentage of participants that agreed to each of the 10 items comprising the Stigma Experiences Scale for each diagnostic group, including reliability coefficients and mean scale scores. Almost all of the scale items were endorsed by a third or less of the participants, and the most frequently endorsed item (avoiding situations that might be stigmatizing, which was endorsed by more than half of the participants) was approximately the same for each diagnostic group. Moreover, the item ‘The expectation that the average person would be afraid of someone with a serious mental illness’ was endorsed by 48% of bipolar patients and 41.9% of depressive patients.

**Table 2:** Reliability coefficients for the 10-item Stigma Experience Scale

Scale item	Bipolar disorder (*n* = 50)		Depression (*n* = 43)	
	% agreed	Item-rest correlation	% agreed	Item-rest correlation
Do you think people will think less of you if they know you have a mental illness?	28%	0.328	41.9%	0.582
Do you think that the average person is afraid of someone with a serious mental illness?	48%	0.429	41.9%	− 0.086
Have you ever been teased, bullied, or harassed because you have a mental illness?	34%	0.380	41.9%	0.476
Have you felt that you have been treated unfairly or that your rights have been denied because you have a mental illness?	26%	0.421	23.3%	0.461
Have your experiences with stigma affected your recovery?	20%	0.441	27.9%	0.622
Have your experiences with stigma caused you to think less about yourself or your abilities?	30%	0.223	27.9%	0.552
Have your experiences with stigma affected your ability to make or keep friends?	20%	0.416	30.2%	0.611
Have your experiences with stigma affected your ability to interact with your family?	24%	0.354	23.3%	0.437
Have your experiences with stigma affected your satisfaction with or quality of life?	36%	0.260	32.6%	0.450
Do you try to avoid situations that may be stigmatizing to you?	56%	0.292	51.2%	0.619
Cronbach’s alpha reliability coefficient	0.692		0.792	
Mean scale score (SD)	3.22 (2.359)		3.418 (2.779)	
95% CI	2.549–3.890		2.563–4.274	

The Cronbach’s alpha of the Stigma Experience Scale was 0.79. The item-rest correlations showed that one item (the extent to which the average person is believed to be afraid of someone with a mental illness) was potentially “problematic”, but only among people with depression (item-rest correlation = − 0.086). However, the Kuder-Richardson coefficients indicated that this subscale produced internally consistent data in both groups—well above the minimum conventional cut-off point of 0.70. The internal consistency of the scale among those with depression improved when this item was removed (KR-20 = 0.84). Even so, this item was retained for theoretical and practical reasons. The mean scale scores were not significantly different between the two groups, which suggests that similar types of stigma experiences occurred within each group.

### The impact of stigma on personal and family Life

Table 3 summarizes the mean item scores and reliability coefficients for the 7-item Stigma Impact Scale. The mean item scores were not significantly different between the bipolar disorder and depression groups. The Cronbach’s alpha of the Stigma Impact Scale was 0.88.

**Table 3:** Reliability coefficients for the 7-item Stigma Impact Scale

Scale item	Bipolar disorder (*N *=50)		Depression (*N *=43)	
	Mean (SD)	Item-rest correlation	Mean (SD)	Item-rest correlation
On a scale where 0 is the lowest possible amount and 10 is the highest possible amount, how much has stigma affected you personally?				
Quality of life	3.4 (3.446)	0.625	3.4 (3.626)	0.60
Social contacts	2.78 (3.824)	0.692	2.72 (3.68)	0.697
Family relations	2.6 (3.73)	0.650	2.81 (3.76)	0.772
Self-esteem	2.86 (3.33)	0.592	2.95 (3.879)	0.760
On a scale where 0 is the lowest possible amount and 10 is the highest possible amount, how much has stigma affected your family as a whole?				
Quality of life	2.58 (3.447)	0.589	1.58 (3.072)	0.567
Social contacts	1.68 (3.09)	0.716	0.84 (2.17)	0.433
Family relations	1.54 (2.887)	0.818	0.74 (2.15)	0.471
Cronbach’s alpha reliability coefficient	0.878		0.851	
Mean scale score (SD)	17.44 (18.14)		15.047 (16.609)	
CI	12.284–22.596		9.935–20.158	

**Fig. 1:**
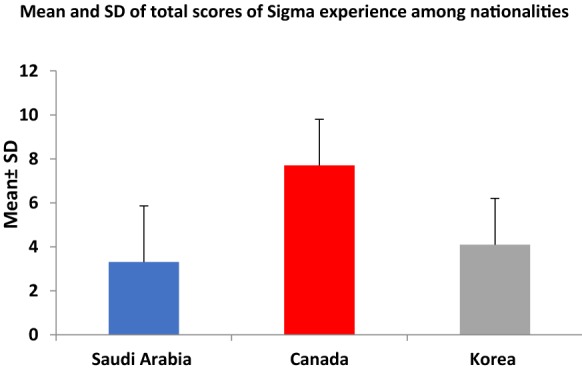
Mean and SD of the total scores for stigma experiences among nationalities

**Fig. 2:**
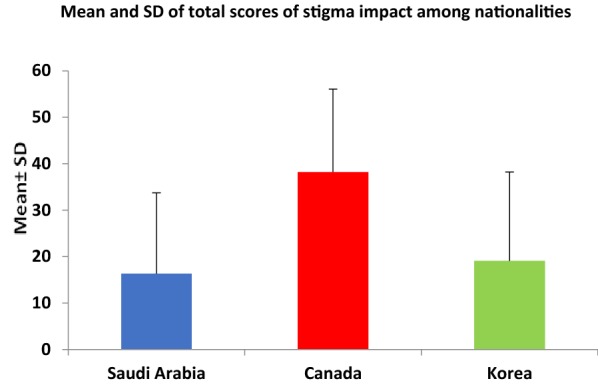
Mean and SD of the total scores for stigma impacts among nationalities

Table 4 summarizes the results of the independent samples t-test by nationality. The difference between Canada and Saudi Arabia was highly significant (*P* < 0.0001; Figs. 1, 2). Regarding all the items on the Stigma Experiences Scale, the Canadians had higher levels of agreement than the Saudis. This difference was highly significant (*P* < 0.0001) except for the item related to being teased, bullied, or harassed because of having a mental illness (*P* = 0.02). Similarly, average scores for each item of the Stigma Impact Scale were all significantly higher in Canadian subjects that Saudi subjects (*P* < 0.0001).

**Table 4:** Independent samples *t*-test (by nationality)

Nationality	Mean (SD)	Mean difference	*P* value
Sum of stigma experience			
Saudi Arabia (n = 93)	3.312 (2.549)		
Canada (n = 54)	7.7 (2.1)	− 4.388	0.0001
Korea (n = 34)	4.1 (2.1)	− 0.788	0.0616
Sum of stigma impact			
Saudi Arabia (n = 93)	16.33 (17.398)		
Canada (n = 54)	38.2 (17.9)	− 21.87	0.0001
Korea (n = 34)	19.1 (19.1)	− 2.77	0.379

Moreover, the patients frequently suggested that they made efforts to increase public awareness. More than one-third of the participants in this study had tried to reduce stigma by educating their friends or family about their psychiatric disorder. Indeed, one-quarter of the participants were motivated by their experiences with stigma to speak out about the rights of mentally ill people. Some participants even suggested various strategies for raising public awareness of the need to reduce the stigma associated with mentally ill people (e.g., educating patients about addressing stigma, educating the public about the difference between mood disorders and psychotic disorders, educating families about the need to include people with psychiatric disorders, and conducting awareness campaigns in schools on a nationwide basis).

## Discussion

This study reports three main findings. First, regarding the Stigma Experience Scale, one item was endorsed by more than half of the Saudi participants with a mood disorder: ‘avoiding situations that might be potentially stigmatizing’. Second, the Saudi population demonstrated the lowest stigma experience and impact compared with both the Canadian and Korean populations. Finally, the Inventory of Stigmatizing Experiences was deemed to be a reliable tool in Saudi patients.

More than half of all the Saudi participants reported trying to hide their mental illness from others and to avoid situations that might lead to being stigmatized. This suggests that self-stigmatization is a pervasive phenomenon. Another explanation could be the excess protection of Saudi participants’ privacy as a result of deeply held traditional customs.

Despite the apparent stigma that was revealed in the review of the literature from Saudi Arabia, the second finding of this study was that, of the three location groups, the Saudi group had the lowest scores on both the Stigma Experience Scale and the Stigma Impact Scale. These findings suggest that there might be higher levels of stigma in Canada and Korea than in Saudi Arabia, but no significant differences were found with the Korean group. This could be explained by the effect of differences in living circumstances, the time interval from when symptoms first presented to the initiation of treatment, involuntary hospitalization, and the current mental health situation among the Canadian, Korean, and Saudi groups.

One-third of the Canadian participants were living alone, compared with only two of the participants from Korea and only one participant from Saudi Arabia. This suggests that there is far more support from the wider community for mentally ill people in Saudi Arabia and Korea than in Canada. It took more than 10 years for 22.6% of the Canadian participants to seek treatment for the first time, whereas only 8.7% and 2.9% of Saudi and Korean participants, respectively, first sought treatment 10 years or more after the onset of their symptoms.

Additionally, a patients’ current mental health situation could affect the level of stigma experienced. The majority of the Saudi and Korean participants reported better current mental health compared to 1 year ago, whereas the majority of the Canadian participants reported that their mental health was generally the same as or worse than it was 1 year previously. These findings might reflect the involvement of Asian families in patient care, which could potentially decrease the level of stigma from the perspective of the patient. Family involvement means that the patient is not usually left to live alone and might also serve as a ‘driving force’ for patients to seek medical treatment. Alternatively, this might lead to patients being hospitalized against their will, thus resulting in better management of their mental health situation. Indeed, there was also a difference in the rate of involuntary hospitalization. Among those who were hospitalized for a mental illness or suicide attempt, 75% of the Saudi sample was hospitalized involuntarily, compared with only 3.6% of the Canadian sample. However, the impact of compulsory admission to psychiatric inpatient treatment can be experienced as self-stigmatizing, which was independently predicted by the experience of higher levels of shame, self-contempt, and stigma-related stress, which might lead to further self-stigmatization, reduced empowerment, and poor quality of life [22].

Finally, our findings yielded reliability coefficients of 0.79 for the Stigma Experiences Scale and 0.88 for the Stigma Impact Scale. This result was similar to that of the Korean study. Thus, the ISE is a reliable tool that can be used to compare stigma experiences and their impact on people with a mood disorder from different cultural backgrounds. ISE might therefore be applicable to Middle Eastern populations and Muslim countries.

### Limitations

Our findings regarding the comparison of Stigma Inventory Scale scores might have been affected by the sample size. This analysis included data from only 54 Canadian and 34 Korean participants, compared to 93 Saudi participants. This restricted sample size might have decreased the power of the analysis.

Another possible explanation for the differences in social desirability bias is that data were collected through face-to-face interviews. This means that what the patients reported might differ from how they might behave under real conditions, because they might have been influenced by the desire to act in a “socially desirable” way. Saudi Arabia is a collectivist society that emphasizes the importance of maintaining relationships with others. Therefore, it might not have been socially desirable for the Saudi participants to express all of their personal experiences with stigma and its impact on their lives.

Additionally, a convenience sampling method was used, which introduces a risk of bias because of the underrepresentation of the population. However, convenience sampling is a cost-effective method for rapidly targeting a sample and easily gathering focused information, which was necessary as we had a limited number of patient volunteers available for face-to-face interviews. Finally, although patients with severe psychosis (e.g., paranoid delusion) were excluded, the participants may still have had paranoid traits that could be associated with negative interpretation bias [23].

## Conclusions

We conclude that more than half of the Saudi participants with a mood disorder avoided situations that might be potentially stigmatizing. Based on our analyses, there are higher patient-reported levels of stigma in Canada and Korea than in Saudi Arabia. Cultural differences and family involvement in patient care could significantly impact self-stigmatization among people with mood disorders. Additionally, the ISE was deemed to be a reliable tool.

Based on our findings, we recommend conducting future studies to address stigma using larger patient sample sizes. Additionally, family support programs could help to reduce stigmatization, which could consequently increase compliance. We suggest examining the attitudes of patients’ families in greater detail.

## Abbreviations

ISE: Inventory of Stigmatizing Experiences
DALYs: disability-adjusted life years
DSM-IV-TR: Diagnostic and Statistical Manual of Mental Disorders-Fourth Edition (Text Revision) (American Psychiatric Association)
KR-20: Kuder–Richardson Formula 20
